# Carbonic anhydrase-8 gene therapy inhibits the ITPR1-cytosolic free calcium pathway producing analgesia and anti-hyperalgesia

**DOI:** 10.1186/1744-8069-10-S1-O7

**Published:** 2014-12-15

**Authors:** Roy C Levitt, Gerald Z Zhuang, Benjamin Keeler, Jeff Grant, Laura Bianchi, Eugene S Fu, Yan Ping Zhang, Diana M Erasso, Jian Guo Cui, Tim Wiltshire, Qiongzhen Li, Shuanglin Hao, Konstantinos D Sarantopoulos, Keith Candiotti, Shad B Smith, William Maixner, Luda Diatchenko, Eden R Martin

**Affiliations:** 1 Department of Anesthesiology, Perioperative Medicine and Pain Management, University of Miami Miller School of Medicine, Miami, FL 33136, USA; 2 Department of Physiology and Biophysics, University of Miami Miller School of Medicine, Miami, FL 33136, USA; 3 Department of Pharmacology and Experimental Therapeutics, Eshelman School of Pharmacy, University of North Carolina, Chapel Hill, NC 27599, USA; 4 Algynomics Inc., Chapel Hill, NC, 27599, USA; 5 John P. Hussman Institute for Human Genomics, University of Miami Miller School of Medicine, Miami, FL, 33136 USA; 6 John T. Macdonald Foundation Department of Human Genetics, University of Miami Miller School of Medicine, Miami, FL, 33136 USA; 7 Bruce W. Carter Miami Veterans Healthcare System, Miami, FL 33136, USA

## Background

Calcium dysregulation is linked with various forms of neuropathology including seizure disorders, multiple sclerosis, Huntington’s disease, Alzheimer’s, spinal cerebellar ataxia (SCA) and chronic pain. Carbonic anhydrase-8 (Car8) is an allosteric inhibitor of inositol trisphosphate receptor-1 (ITPR1), which regulates intracellular calcium release fundamental to critical cellular functions including neuronal excitability, neurite outgrowth, neurotransmitter release, mitochondrial energy production, cell fate and neuroplasticity through the regulation of transcription and protein synthesis. Herein, we test the hypothesis that Car8 regulation of ITPR1 and cytoplasmic free calcium release is critical to nociception and pain behaviors, and a potential therapeutic target for persistent pain.

## Materials and methods

The homozygous ‘*waddles’* (*wdl*) mouse is a Car8 null mutant (MT) due to a 19 Bp deletion in exon 8 that occurred spontaneously in the C57BLKS (WT) background producing a truncated unstable protein. MT and WT mice were purchased from Jackson Labs. Behavioral testing was conducted using von Frey filaments and Hargreaves methods as previously described.[1,2] AAV2 vectors were constructed from WT cDNA (ATCC) and Car8 was mutagenized to produce the 19 Bp deletion (negative control) using site directed mutagenesis, and WT and MT constructs were packaged in AAV8 viral particles. Car8 AAV-mediated gene transfer (<1E+14/1.5 microL) of WT (*V5-mCar8WT*) and MT (control *V5-mCar8MT*) via sciatic nerve injections was used to rescue nociceptor hypersensitivity in *wdl* mice. Immunostaining, westerns, RT-PCR, and calcium imaging were performed as described previously.[3,4]

## Results

We show that the homozygous ‘*waddles’* (*wdl*) MT mice exhibiting mechanical allodynia and thermal hyperalgesia compared to WT mice. Dorsal root ganglia (DRG) from MT mice also demonstrate increased steady-state ITPR1 phosphorylation (pITPR1) and cytoplasmic free calcium release compared to WT mice. Overexpression of V5-Car8WT protein in MT nociceptors complements Car8 deficiency, down regulates pITPR1, and abolishes thermal hyperalgesia and mechanical allodynia. We further demonstrate that inflammation down regulates Car8 nociceptor expression, producing a deficiency relative to ITPR1, and increased pITPR1 in WT mice as a potential mechanism of hypersensitivity and calcium dysregulation. Finally, nociceptor gene transfer of V5- Car8WT (but not V5-Car8MT gene transfer) produces analgesia and anti-hyperalgesia in subacute and chronic inflammatory pain models.

## Conclusions

Our findings indicate Car8 controls an intracellular calcium-regulating pathway critical to nociception, inflammatory pain and possibly other neuropathological states. Car8 and ITPR1 represent important new targets for persistent pain conditions. Herein we provide a proof-of-concept for Car8 nociceptor directed gene therapy for inflammatory pain.
